# An Atypical Case of Chronic Fungal Rhinosinusitis: Temporary Symptom Relief Resulted in a Delay of Diagnosis and Brain Abscess

**DOI:** 10.7759/cureus.82226

**Published:** 2025-04-14

**Authors:** Mio Yamamoto, Takeshi Takahashi, Ryota Kai, Tetsuhisa Hatase, Arata Horii

**Affiliations:** 1 Otolaryngology - Head and Neck Surgery, Niigata University Hospital, Niigata, JPN; 2 Otolaryngology - Head and Neck Surgery, Niigata University, Niigata, JPN; 3 Ophthalmology, Niigata University Hospital, Niigata, JPN

**Keywords:** aspergillus, brain abscess, diabetes mellitus, invasive fungal rhinosinusitis, orbital apex syndrome

## Abstract

A 67-year-old diabetic man with chronic invasive fungal rhinosinusitis (IFRS) experienced mild, remitting visual symptoms, delaying diagnosis. Despite early MRI evidence of orbital apex inflammation, spontaneous symptom resolution led to discontinuation of hospital visit. Eleven months later, he developed orbital apex syndrome and intracranial complications. Emergency surgery and antifungal therapy were initiated, but vision loss persisted. This case underscores the risk of transient symptom remission masking IFRS progression, emphasizing the importance of early intervention.

## Introduction

The most commonly accepted diagnostic system divides fungal rhinosinusitis into two groups: invasive and noninvasive according to the histopathological findings. Invasive fungal rhinosinusitis (IFRS) is further divided into acute and chronic by the disease onset: acute IFRS has a time course of four weeks or less, whereas chronic IFRS is categorized as having a time course of >12 weeks [1]. Regarding chronic IFRS, a systematic review published in 2024, reported that 1) the chief symptoms were visual change, headache, and facial pain, 2) the average duration of symptoms was 6.47 months, and 3) the rate of mortality during follow-up was 12% [2]. Chronic IFRS mainly affects immunocompromised patients, including those with diabetes or organ transplant recipients, which usually takes a progressive and aggressive course without remission.

In this report, we describe a rare case of chronic IFRS, which showed a spontaneous remission of visual symptoms, resulting in a delay of diagnosis/treatment and serious intracranial complications such as brain abscess.

## Case presentation

**Figure 1:**
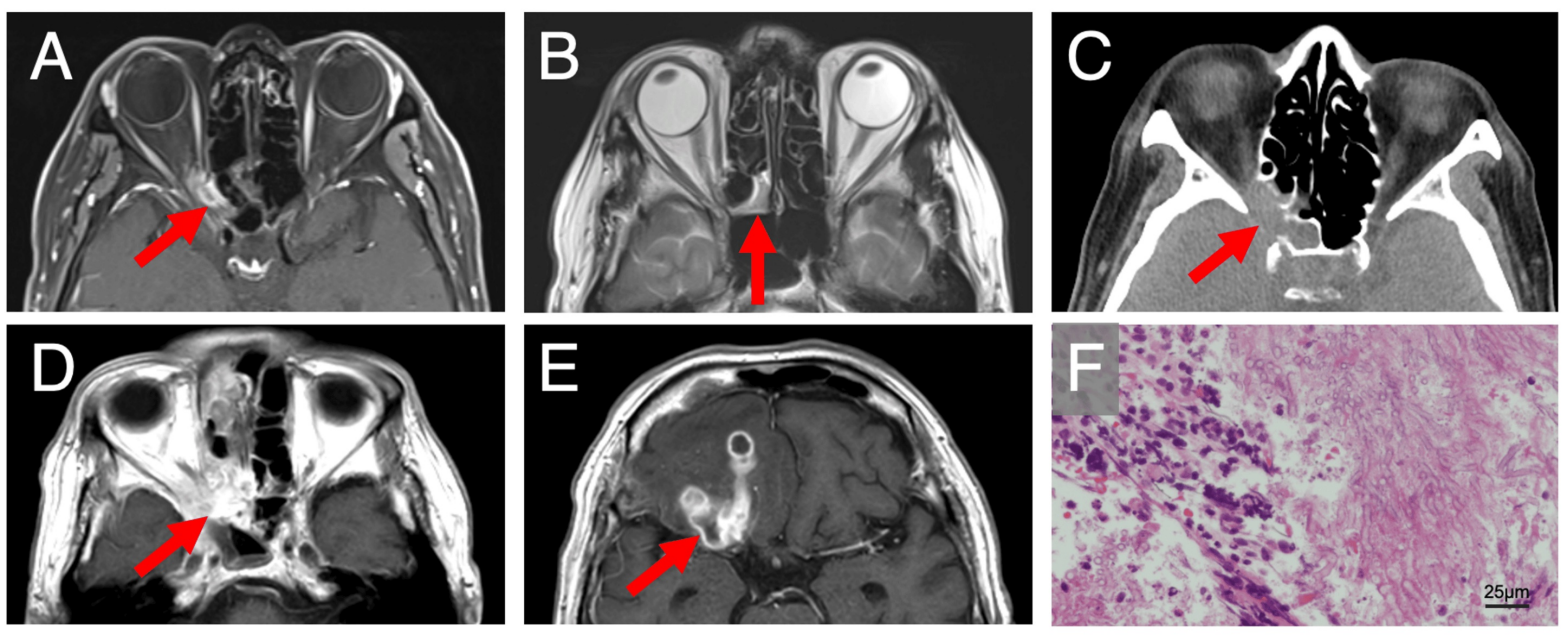
Imaging findings. A, B) Contrast-enhanced T1 and T2-weighted images of MRI 11 months prior to brain abscess formation. Inflammation in the right orbital apex (A) with mucosal thickening in the posterior ethmoid sinus (B) was observed. C) Non-contrast enhanced CT scan at the onset of brain abscess. This CT scan revealed a soft tissue density with bone destruction (arrow) extending from the right posterior ethmoid sinus to the orbital apex. D, E) Contrast-enhanced T1-weighted images of MRI at the onset of brain abscess. This MRI revealed a well-enhanced lesion in the right posterior ethmoid sinus (D) and multiple ring-enhanced lesions (arrow) in the right frontal lobe (E). F) Pathological examination. This examination revealed an Aspergillus specimen.

A 67-year-old man with a history of type II diabetes mellitus visited the ophthalmology department with a two-month history of mild visual loss in the right eye and intermittent retro-orbital pain (14 months prior to our admission). He had visited the ophthalmology clinic; his visual acuity was 20/50 in the right eye. The intraocular pressure was within normal range. Anterior segment and fundus showed no findings of note that would cause visual acuity loss. Therefore, a brain MRI was performed at a neurosurgery clinic to seek central lesions that would explain the patient’s symptoms. However, no abnormal findings were identified. His ocular symptoms alternated between temporary improvement and exacerbation. Therefore, he was referred to a neuro-ophthalmologist in the Department of Ophthalmology at a university hospital for further evaluation of the symptoms (11 months prior to our admission). His visual acuity was 20/40 on the right and 20/20 on the left. The intraocular pressure was 11 mmHg on the right and 14 mmHg on the left. The pupil was isocoria and a round shape. He exhibited orthophoria, and his eye movements were normal. An anterior segment examination revealed an incipient cataract, a fundus examination showed only mild non-proliferative diabetic retinopathy, and the optic disc was normal. These findings were insufficient to explain his vision loss. A relative afferent pupillary defect was observed in the right eye. MRI revealed inflammation in the right orbital apex (Figure 1A) and mucosal thickening in the posterior ethmoid sinus (Figure 1B). Based on these results, the ophthalmologist considered the possibility of right rhinogenous optic neuropathy and decided to refer the patient to the Department of Otolaryngology. However, the patient discontinued hospital visits without treatment because the symptoms resolved spontaneously. Over several months from that time, his blood sugar was well-controlled for unknown reasons, as indicated by a marked decrease in glycated hemoglobin (HbA1c) level. Eleven months later, he presented to the emergency department with a complaint of mild altered consciousness, accompanied by progressive visual loss and intermittent retro-orbital pain once again over the past month. Physical examination revealed that his right eye exhibited light perception only, mild myosis, loss of pupillary light reflex, eyelid ptosis, and outward deviation - all consistent with orbital apex syndrome (OAS). The key blood test results are shown in Table 1.

**Table 1:** The key blood test results PG: plasma glucose; HbA1c: hemoglobin A1c; NGSP: National Glycohemoglobin Standardization Program

Parameter (unit)	Value	Reference range
White blood cell count (10^2^/μL)	145	35-98
Neutrophils (%)	88.5	42-74
C-reactive protein (mg/dL)	7.78	0-0.14
Blood glucose (PG) (mg/dL)	130	70-110
HbA1c (NGSP) (%)	5.9	4.6-6.2

CT revealed a region of soft tissue density with bone destruction extending from the right posterior ethmoid sinus to the orbital apex (Figure 1C). Contrast-enhanced MRI revealed a well-enhanced lesion in the right posterior ethmoid sinus (Figure 1D) and multiple ring-enhancing lesions in the right frontal lobe (Figure 1E), leading to a diagnosis of a brain abscess secondary to sino-orbital infection. The patient underwent emergency endoscopic sinus surgery, after which meropenem 6 g/day, vancomycin 1.5 g/day, and voriconazole 600 mg/day were administered immediately. Fungal culture of the sinus specimen yielded Aspergillus species, and pathological examination revealed an Aspergillus specimen (Figure 1F). Based on these findings, a diagnosis of IFRS was made. The patient received combined antibacterial and antifungal treatment until postoperative day 17, followed by antifungal therapy alone. After gradual recovery, he was discharged on postoperative day 74. He has since been followed for five months with tapered oral voriconazole, regaining daily life activities, but remains blind in the right eye.

## Discussion

**Figure 2:**
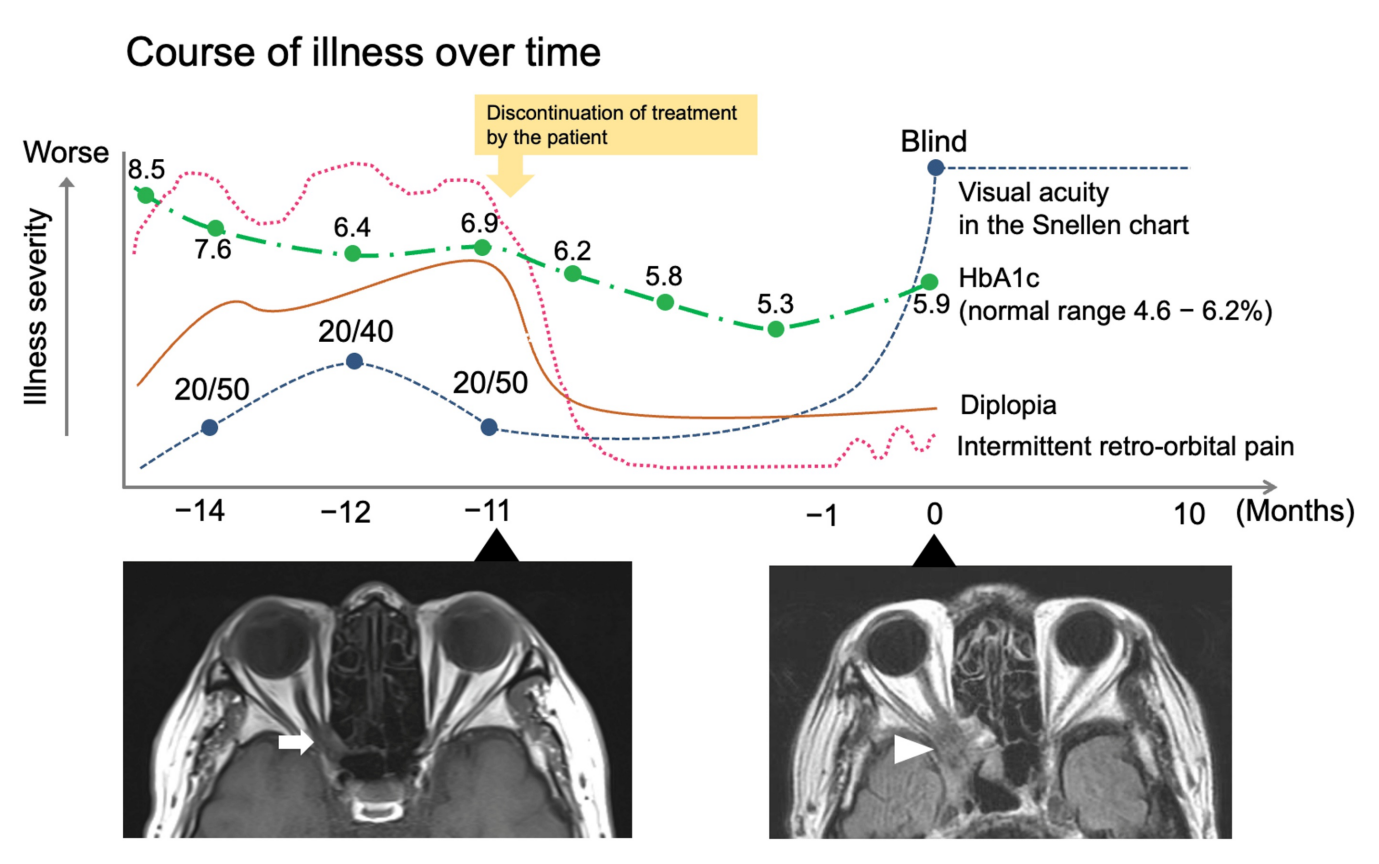
The course of illness and MR images over time The upper panel shows the course of illness. T2-weighted image of an MRI scan performed 11 months prior to the onset of the brain abscess revealed an optic nerve deviation but no prominent infiltration (arrow). In contrast, an MRI scan at the onset of the brain abscess revealed distinct infiltration (arrowhead).

Herein, we report a case of a brain abscess and OAS due to IFRS in which symptoms first appeared approximately 14 months prior, followed by spontaneous improvement without medical intervention. Although an MRI performed at the university hospital pointed an inflammation in the right orbital apex (Figure 1A) and mucosal thickening in the posterior ethmoid sinus (Figure 1B), the patient discontinued hospital visits because of his symptom relief, resulting in the later development of a brain abscess approximately 11 months later. Figure 2 shows his course of illness over time.

The typical course of OAS due to IFRS is characterized by rapid progression over a few days or weeks in patients who are immunocompromised, including those with diabetes mellitus [3]. Our patient presented with an atypical chronic and remitting progressive course. Given that his HbA1c level was favorable at 5.9% at his visit to our department, we suspected that the chronic course of the disease may have been attributable to subtle changes in immune status, potentially leading to atypical patterns of disease progression [4]. The prognosis of OAS-associated IFRS remains poor, as reported in a 2021 review, with 32% of cases resulting in death [5]. In patients suspected of having OAS secondary to IFRS, immediate empirical antifungal therapy and tissue biopsy are essential [6]. As relapse and fatality have been reported after a prolonged disease course, close follow-up is also warranted [7]. Chronic but not acute IFRS may exhibit an atypical course of OAS in patients with well-controlled diabetes mellitus. Even in cases of suspected OAS that spontaneously remits, the condition may relapse with rapid progression. Therefore, aggressive treatment is essential as soon as the initial manifestations of IFRS appear to prevent vision loss.

## Conclusions

This case highlights the atypical presentation and progression of chronic IFRS in a patient with well-controlled diabetes mellitus. Transient symptom remission led to a delay in diagnosis and treatment, ultimately resulting in irreversible visual loss and the development of a brain abscess. Clinicians should remain vigilant even when symptoms appear to improve spontaneously, as early intervention is crucial to prevent severe complications associated with IFRS.
